# Isolation of cell type-specific apoptotic bodies by fluorescence-activated cell sorting

**DOI:** 10.1038/srep39846

**Published:** 2017-01-06

**Authors:** Georgia K. Atkin-Smith, Stephanie Paone, Damien J. Zanker, Mubing Duan, Than K. Phan, Weisan Chen, Mark D. Hulett, Ivan K. H. Poon

**Affiliations:** 1 Department of Biochemistry and Genetics, La Trobe Institute for Molecular Science, La Trobe University, Melbourne, Victoria 3086, Australia

## Abstract

Apoptotic bodies (ApoBDs) are membrane-bound extracellular vesicles that can mediate intercellular communication in physiological and pathological settings. By combining recently developed analytical strategies with fluorescence-activated cell sorting (FACS), we have developed a method that enables the isolation of ApoBDs from cultured cells to 99% purity. In addition, this approach also enables the identification and isolation of cell type-specific ApoBDs from tissue, bodily fluid and blood-derived samples.

ApoBDs are the largest type of extracellular vesicle (typically 1–5 μm in diameter) generated through a process termed apoptotic cell disassembly^1^. As ApoBDs can harbour DNA, microRNA and proteins, the disassembly of an apoptotic cell into ApoBDs can mediate intercellular communication through the transport of these biomolecules and may contribute to the development of various disease states^2^,^3^. For example, endothelial cell-derived ApoBDs containing the cytokine IL-1α can induce chemokine secretion and mediate sterile inflammation^2^. Furthermore, DNA and nuclear proteins (e.g. histones, La/SSB) can be transferred to surrounding phagocytes via ApoBDs and may contribute to the progression of autoimmune diseases like systemic lupus erythematosis^3^,^4^,^5^. Although the importance of ApoBD formation in pathological settings including inflammation^2^, autoimmunity^3^,^4^,^5^, tumourigenesis^6^, and viral infection^7^ is emerging, the characterisation of ApoBD content and function is not fully defined due to the lack of a high purity isolation technique. Therefore, we aimed to develop a method to isolate ApoBDs to high purity based on specific biological characteristics of ApoBDs, and to identify and purify cell-type specific ApoBDs from complex biological samples.

For most studies, ApoBDs have been isolated using a differential centrifugation approach. These methods (denoted here as the traditional differential centrifugation approach) often include an initial centrifugation at 300–500 *g* to pellet cells, followed by centrifugation of resultant supernatant at 1,000–4,000 *g* to pellet ApoBDs^2^,^8^,^9^ (Fig. 1a). Although such methods can enrich ApoBDs to ~84% purity (Fig. 1b), the initial 300–500 *g* centrifugation may pellet larger ApoBDs and exclude them from subsequent analysis. To overcome this, we recently demonstrated that by performing a series of low-speed centrifugation steps, ApoBDs can also be isolated from cells to ~83% purity (Fig. 1a,c)^1^. Although differential centrifugation approaches are the current gold standard for the isolation of ApoBDs, such methods are designed to purify ApoBDs based solely on density rather than known biological characteristics. Additionally, the presence of cells in ApoBD preparations may influence the outcome of downstream analyses (such as microRNA and proteomic analysis). Furthermore, ApoBDs derived from different cell types cannot be isolated independently via differential centrifugation approaches and therefore, the respective functions and contents of cell type-specific ApoBDs remain poorly understood. To overcome these limitations, we developed a FACS-based approach that allows for detection and isolation of ApoBDs based on characteristics including particle size, granularity and phosphatidylserine (PS) exposure.

Recently, we established a cell staining and flow cytometry approach that can identify ApoBDs from viable, apoptotic and necrotic cells based on relative size and granularity, in addition to differential TO-PRO-3 and annexin A5 (A5) staining^1^,^10^,^11^. By further adapting this analytical method, we can use FACS to isolate ApoBDs from a cell culture sample. Using apoptotic (UV irradiated) THP-1 monocytes as a model, we found that after enrichment of ApoBDs by low-speed centrifugation to ~83% purity, ApoBDs could be further purified from remaining cells through FACS to 99% purity (Fig. 1c). Furthermore, ApoBDs were also isolated directly from a whole apoptotic sample (WAS) by FACS to 99% purity, indicating that prior enrichment is not required and FACS alone is sufficient to obtain a highly pure population of ApoBDs (Fig. 1d). Consistent with previous studies, ApoBDs isolated via FACS exhibited intermediate TO-PRO-3 and A5 staining^1^,^10^,^11^ (Fig. 1e, Supplementary Fig. 1). For further validation of purity, sorted ApoBD samples were visualised by differential interference contrast (DIC) microscopy which demonstrated that all ApoBDs isolated were subcellular fragments, thus within the expected size range for ApoBDs generated by human monocytic cells (Fig. 1f,g). Importantly, to avoid co-isolation of microparticles (pelleted at ~12,000 *g*, 0.1–1 μm,) and exosomes (pelleted at ~120,000 *g,* 0.2 μm)^9^,^12^, two 1,000 *g* centrifugation steps are performed during sample preparation to pellet ApoBDs for FACS (Fig. 1a). Additionally, the FSC sensitivity of the BD-FACS Aria III (used for FACS-based purification) detects particles greater than 0.5 μm, further limiting analysis specifically to ApoBDs (BD FACS Aria III Technical Specification, BD Biosciences). Taken together, these results indicate that by using a FACS-based approach, ApoBDs can be isolated directly from a WAS to high purity.

To ensure that ApoBDs isolated via FACS will maintain plasma membrane integrity for subsequent functional analysis, we monitored uptake of the membrane impermeable dye propidium iodide (PI) by ApoBDs via confocal microscopy^13^. Consistent with previous studies^13^, a subset (approximately 30%) of ApoBDs undergo membrane lysis in culture (Fig. 1h). However, there was no additional loss in ApoBD membrane integrity after purification via FACS (Fig. 1h), indicating that ApoBDs can be isolated by a FACS-based approach without compromising plasma membrane integrity. In addition to THP-1 monocytes, this FACS-based approach can also be adapted to the purification of ApoBDs generated by other cell types including human Jurkat T cells and primary human umbilical vein endothelial cells (HUVEC), which were purified to 96 and 99% purity, respectively (Fig. 1i,j). Together, these results demonstrate that a FACS-based approach enables efficient isolation of intact ApoBDs directly from a cell culture-derived WAS, with greater purity compared to previous centrifugation techniques.

Recent studies have demonstrated that apoptotic cell disassembly occurs *in vivo*^14^,^15^ and is implicated in the pathogenesis of various diseases^3^. Furthermore, as ApoBDs generated from different cell types may play distinct functional roles^1^,^16^, the isolation of cell type-specific ApoBDs is important to gain insight into various disease states. Therefore, we asked whether this FACS-based approach could also be used to isolate cell type-specific ApoBDs *ex vivo*. Initially, we examined the cell disassembly process in the thymus as dexamethasone treatment can induce CD4^+^/CD8^+^ thymocyte apoptosis^17^, and apoptotic thymocytes generate an abundance of ApoBDs^11^ (Fig. 2a). To confirm the origin of ApoBDs, we monitored cell type-specific markers (i.e. CD4 and CD8) in combination with size, granularity, TO-PRO-3 uptake and A5 staining. Furthermore, thymocyte-derived ApoBDs from a single-cell suspension generated from the thymus were purified from remaining cells to ~93% purity (Fig. 2b). Importantly, ~85% of the sorted ApoBDs exhibited intermediate CD4/CD8 staining, confirming thymocyte origin (Fig. 2c,d, Supplementary Fig. 2) and demonstrates that ApoBDs derived from a specific cell type can be isolated from a complex tissue sample.

Next, we investigated whether this FACS-based approach could be used to isolate ApoBDs in a disease setting. To address this, we utilised an influenza A virus (IAV) infection mouse model as IAV induces both macrophage and monocyte apoptosis^18^ (Fig. 2e). ApoBDs from bronchoalveolar lavage (BAL) were first identified by CD45 staining to confirm immune cell origin, and then by Ly6G to exclude neutrophils and neutrophil-derived ApoBDs. Next, monocyte and macrophage-derived ApoBDs were identified based on differential CD64 (monocyte marker) and SiglecF (alveolar macrophage marker) staining (Supplementary Fig. 3). When using the FACS-based approach to sort monocyte-derived ApoBDs, total ApoBDs were purified from remaining BAL cells to ~86% purity (Fig. 2f). Importantly, when comparing the percentage of macrophage, monocyte and neutrophil-derived ApoBDs within the post-sort sample, 98% exhibited intermediate CD64 staining, confirming monocytic origin (Fig. 2g,h). Purity quantification however does not include ApoBDs negative for cell type-specific markers as this could be due to a loss or bleaching of fluorophores on ApoBDs through re-analysis by flow cytometry.

Finally, to determine if the FACS-based approach could be adapted to isolate ApoBDs from human patient samples, we isolated ApoBDs from a WAS derived from human peripheral blood mononuclear cells (PBMCs) (Fig. 2i). PBMCs were first purified from whole blood of healthy donors and exposed to UV irradiation to induce apoptosis. A combination of cell type-specific staining allowed for the differentiation of monocyte and T cell-derived ApoBD subsets (Supplementary Fig. 4). Furthermore, when sorting monocyte-derived (CD14^inter^/CD11b^inter^) and T cell-derived (CD3^inter^) ApoBDs, total ApoBDs were purified from remaining cells to ~61% and ~80% purity, respectively (Fig. 2j). Decreased purity in the sorted monocyte ApoBD sample indicates that the efficiency of the FACS-based approach may differ between cell types from human samples. Nevertheless, 94% of sorted monocyte ApoBDs exhibited intermediate CD14/CD11b staining and only ~3% were of T cell origin (Fig. 2k,l). Similarly, 93% of sorted T cell ApoBDs exhibited intermediate CD3 staining and only ~5% were derived from monocytes (Fig. 2k,l). Like for BAL analysis, purity quantification did not include ApoBDs negative for cell type-specific markers. Collectively, these results demonstrate for the first time that cell type-specific ApoBDs can be isolated using this FACS-based approach.

The formation of ApoBDs is a key downstream process of cell death and has been proposed to regulate a variety of normal physiological and pathological conditions^19^. Previously, ApoBDs have been isolated and studied using differential centrifugation methods based exclusively on density with limited purity. By developing new analytical strategies and using a FACS-based approach, we have shown that ApoBDs can be purified from cell culture samples to high purity. For the first time, we have also demonstrated that by exploiting the unique characteristics of ApoBDs and using cell type-specific markers, ApoBDs from different cell types can be identified and isolated simultaneously from complex biological samples derived from tissue, bodily fluid and blood. Therefore, this FACS-based approach can be adapted for studying ApoBDs generated from model organisms as well as patient samples. Although the FACS-based approach may require lengthy sorting periods to acquire the desired number of ApoBDs, coupling with biochemical and functional assays will provide fundamental insights into the molecular characteristics and functional significance of ApoBD formation.

## Methods

### Reagents and reagent preparation

A5-FITC (556419), A5-V450 (560506), A5-APC (550475), 10x A5 binding buffer (556454), anti-mouse Ly6G APC-Cy7 (560600), anti-mouse SiglecF PE (552126), anti-human CD11b PE (555388), mouse FcR blocking reagent (553141) and anti-human CD45 PE-Cy7 (557748) were purchased from BD Biosciences. TO-PRO-3 iodide (T3605) and PO-PRO-1 (P3581) were purchased from Life Technologies. The following antibodies were purchased from eBioscience; anti-mouse CD4 PE-Cy7 (25-0042-82), anti-mouse CD8a PE (12-0081-82), anti-human CD3 APC-Cy7 (17-0031-83) and anti-human CD56 Pe-Cy5 (15-0567-42). Anti-mouse CD45.2 FITC (109806) and anti-mouse CD64 PE-Cy7 (139313) were purchased from BioLegend. Anti-human CD14 FITC (130-098-063) and human FcR blocking reagent (130-059-901) were purchased from MAC Miltenyi Biotech. Ficoll Paque Premium was purchased from GE Healthcare Life Science (17-5442-02). Complete media was prepared using RPMI 1640 medium (Life Technologies, 22400-089), 50 IU ml^−1^ penicillin and 50 μg ml^−1^ streptomycin mixture (Life Technologies, 15140122), 10% (vol/vol) fetal calf serum (FSC) (Gibco, 10099-141) and 0.2% (vol/vol) MycoZap (Lonza, VZA-2012). FACS buffer was prepared using 10% FCS, 2x A5 binding buffer and 2 mM EDTA (Sigma, 1001710526) in PBS.

### Cell culture

THP-1 monocytic and Jurkat T cell lines were obtained from ATCC and cultured in complete RPMI. HUVECs were obtained from Lonza and cultured in Clonetics^TM^ EGM^TM^-2 BulletKit^TM^ containing EBM-^TM^ basal medium and the following growth supplements: human epidermal growth factor (hEGF), vascular endothelial growth factor (VEGF), R3- insulin-like growth factor-1 (R3-IGF-1), ascorbic acid, hydrocortisone, human fibroblast growth factor-beta Hfgf-β, heparin, fetal bovine serum (FBS) and genatmicin/amphotericin-B (GA). All cell lines were cultured at 37 °C, 5% CO_2_.

### Mice

For dexamethasone treatment, 4–6 week old C57BL/6 mice (male and female) were used under approval of AEC15-36, La Trobe University. For IAV studies, female C57BL/6 mice were purchased from the Walter Eliza Hall Institute of Medical Research (Kew, Melbourne, VIC, Australia). Mice were housed in specific pathogen-free (SPF) isolators and infected with IAV at 6–8 weeks of age under ethics approval of AEC15-85. All methods were carried out in accordance with relevant guidelines and regulations, and all experiments were approved by the La Trobe University Animal Ethics Committee in accordance with the National Health and Medical Research Council Australia code of practice for the care and use of animals for scientific purposes.

### Cell preparation and induction of cell death

For a substantial quantity of ApoBDs to be collected, approximately 5–10 × 10^6^ cells per sample were used. THP-1 monocyte, Jurkat T cell, and HUVEC apoptosis was induced by UV irradiation with a Stratagene UV Stratalinker 1800 (Alient Technologies) at 150 mJ cm^−2^. After apoptosis induction, cells were incubated until the peak time of ApoBD formation (THP-1 monocytes: 2 h, Jurkat T cells: 2.5 h, HUVEC: 6 h,). To induce membrane permeabilisation, ~0.5 × 10^6^ sorted THP-1 cell-derived ApoBDs were exposed to hyperthermic treatment (56 °C) for 1 h.

### FACS staining for single-cell suspension

Although this protocol includes specific stains or fluorescent antibody conjugates, please note that fluorophores can be altered as long as single stain controls are included and appropriate compensation is performed. Apoptotic samples were collected at peak time of ApoBD formation and pelleted at 1,000 *g* for 6 min. Note that only culture supernatant was collected from apoptotic HUVEC samples as viable cells remain adherent. Single-cell culture samples (THP-1, Jurkat, HUVEC) were resuspend in 1 mL of staining solution per 10 × 10^6^ cells containing 1 mL 2x A5 binding buffer, 75 μL A5-FITC and 2 μL TO-PRO-3. Samples were incubated at RT for 10 min and pelleted at 1,000 *g* for 5 min. Samples were resuspended in sterile filtered FACS buffer and filtered through a 70 μm cell strainer prior to FACS. Additionally, samples were kept on ice and in the dark to limit cell death progression, cell lysis or bleaching of fluorescent dyes.

### ApoBD purification by centrifugation

To isolate ApoBDs via a traditional differential centrifugation approach, methods from Berda-Haddad *et al*.^2^, Crescitelli *et al*.^9^ and Lleo *et al*.^8^ were adapted to create a representative method as follows^2^,^8^,^9^. To remove cells, ~5 × 10^6^ apoptotic THP-1 monocytes were collected (2 h post UV irradiation) and centrifuged at 300 *g* for 10 min. The resultant supernatant was centrifuged at 3,000 *g* for 20 min to pellet ApoBDs. A low-speed centrifugation protocol was adapted from Atkin-Smith *et al*.^1^. Firstly, the apoptotic sample was centrifuged at 50 *g* for 5 min to remove cells. The supernatant containing ApoBDs was then collected and centrifuged at 50 *g* for 5 min and repeated twice to remove remaining cells. The final supernatant was centrifuged at 1,000 *g* to pellet ApoBDs. ApoBD sample purity was validated by staining and flow cytometry as outlined above.

### Dexamethasone mouse model

To induce thymocyte apoptosis, 250 μg of dexamethasone in 300 μL PBS was administered by intraperitoneal injection. Mice were euthanized by CO_2_ asphyxiation 6 h post dexamethasone treatment. The thymus was harvested and homogenised through a 70 μm cell strainer in complete RPMI. Samples were pelleted at 1,000 *g* and resuspended in staining solution containing 1 mL 2x A5 binding buffer, 2 μL TO-PRO-3, 10 μL A5-FITC, 10 μL anti-mouse CD4 PE-Cy7 and 10 μL anti-mouse CD8 PE. Samples were incubated at RT for 10 min and pelleted at 1,000 *g* for 5 min. Samples were resuspended in sterile FACS buffer and filtered through a 70 μm cell strainer.

### IAV mouse model

To induce monocyte apoptosis within the lung, mice were infected with 1,000 pfu PR8 (strain A/Puerto Rico/8/1934 H1N1; in 30 μl PBS) intranasally under methoxyflurane-induced anaesthesia. Day 3 post-infection, mice were euthanized with a subcutaneous injection of lethabarb (320 mg ml^−1^). To collect BAL, the trachea was cannulated and 4 × 0.4 mL aliquots of lavage buffer (2% FCS, 2 mM EDTA, PBS) were delivered to and retrieved from the lungs. Cells were pelleted at 1,000 *g* for 5 min and two lavage samples were pooled together. Samples were first incubated in 300 μL 2x A5 binding buffer with 16 μL A5-APC and 2 μl PO-PRO-1 for 10 min at RT. Samples were then pelleted at 1,000 *g* for 5 min and resuspended in 400 μL 2x A5 binding buffer with 1.33 μL FcR block for 10 min on ice. Samples were again pelleted and resuspended in antibody cocktail containing 400 μL 2x A5 binding buffer with 1.33 μL of anti-mouse CD45 FITC, anti-mouse Ly6G APC-Cy7, anti-mouse CD64 PE-Cy7 and anti-mouse SiglecF PE, and incubated for 20 min on ice. Samples were pelleted at 1,000 *g* for 5 min, resuspended in sterile FACS buffer and filtered through a 70 μm cell strainer.

### PBMC model

For PBMC analysis, whole blood was collected from healthy donors (Australian Red Cross Blood Service, agreement number: 14-11 VIC-03, all methods were carried out in accordance with relevant guidelines and regulations, and all experimental protocols were approved by La Trobe University Human Ethics: FHEC09/R16) and diluted 1:3 with 2 mM EDTA, PBS. Solution was layered on a Ficoll Paque gradient and centrifuged for 30 min at 450 *g* at RT. PBMCs were collected and washed 3 times in PBS at 200 *g* for 10 min to remove platelets. Finally, ~40 × 10^6^ PBMCs were collected and exposed to UV irradiation to induce apoptosis as outlined above. Apoptotic cells were collected 3.5 h post UV exposure and stained in 1 mL 2x A5 binding buffer, 50 μL AV-V450 and 2 μL TO-PRO-3 for 10 min at RT then pelleted at 1,000 *g* for 5 min. Samples were resuspended in 1 mL 2x A5 binding buffer with 2 μL FcR block and incubated for 10 min on ice. After pelleting at 1,000 *g*, samples were resuspended in antibody cocktail containing 1,000 μL 2x A5 binding buffer, 20 μL anti-human CD45 Pe-Cy7, anti-human CD11b PE, 10 μL anti-human CD14 FITC, anti-human CD3 APC-Cy7 and anti-CD56 PE-Cy5 and incubated on ice for 20 min. After pelleting at 1,000 *g* for 5 min, samples were resuspended in sterile FACS buffer and filtered through a 70 μm cell strainer.

### FACS and data acquisition

For FACS analysis, FACS Aria III flow cytometer and FACSDiva 6.1.1 software (BD Bioscience) were used. During sorting the flow rate was restricted to <2,000 events/sec to ensure minimal contamination. Additionally, a ‘4 way purity’ sort option was used and is sufficient to gain a 99% pure sample. Although ‘single cell purity’ can be implemented to increase sample purity, acquisition time required to gain desired events increases substantially. Before sorting commenced, appropriate settings were determined for all parameters and compensation was performed. ApoBDs were collected in FACS tubes or 15 mL falcon tubes containing FACS buffer which was necessary to retain A5 staining. Sorting was performed according to the electronic gating strategies provided in Supplementary Figs 1–4. All data was analysed using FlowJo 8.8.6 and 8.8.10 software (Tree Star).

### Confocal microscopy

For microscopy analysis, the Zeiss Spinning Disk and Zeiss LSM 780 confocal microscopes were used at 63× objective and maintained at 37 °C, 5% CO_2_. Samples were imaged in the 8 well Nunc^®^ Lab-Tek^®^ II chamber Slide system. For DIC microscopy images, ~0.5 × 10^6^ ApoBDs were collected after enrichment by low-speed centrifugation or after low-speed centrifugation coupled with FACS then resuspended in 200 μL PBS for microscopy analysis. For PI uptake assays, ~0.5 × 10^6^ sorted ApoBDs, ~1 × 10^6^ WAS and ~0.5 × 10^6^ membrane permeabilised ApoBD samples were subjected to centrifugation at 1,000 *g* for 5 min. Samples were resuspended in 200 μL PBS containing 1 μg mL^−1^ PI for microscopy analysis.

### Statistical analyses

Unless otherwise specified, data is presented as mean ± the standard error of the mean (s.e.m.). To determine statistical significance between two groups (control versus a specific treatment), unpaired Student’s two-tailed *t*-test was applied. A *P* value of less than 0.05 was considered statistically significant.

## Additional Information

**How to cite this article**: Atkin-Smith, G. K. *et al*. Isolation of cell type-specific apoptotic bodies by fluorescence-activated cell sorting. *Sci. Rep.* **7**, 39846; doi: 10.1038/srep39846 (2017).

**Publisher's note:** Springer Nature remains neutral with regard to jurisdictional claims in published maps and institutional affiliations.

## Supplementary Material

Supplementary Information

## Figures and Tables

**Figure 1:**
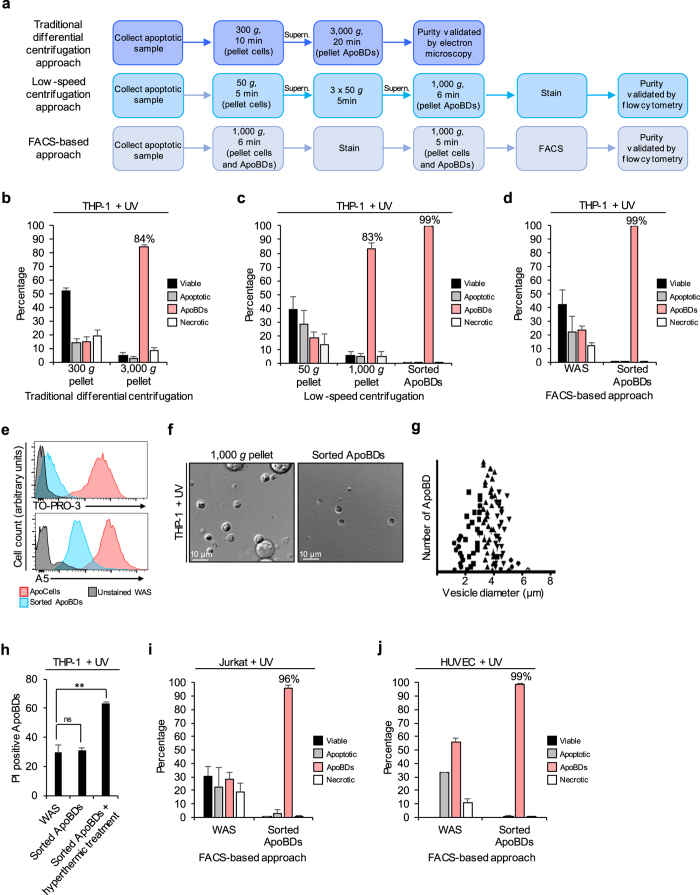
Cell culture-derived ApoBDs can be isolated by FACS to high purity. **(a)** Schematic diagram of ApoBD purification steps for two pre-existing centrifugation approaches, and the newly developed FACS-based approach (supern = supernatant). **(b–d)** Flow cytometry analysis of the purity of THP-1 monocyte-derived ApoBDs isolated by **(b)** traditional differential centrifugation approach, **(c)** low-speed centrifugation approach, and **(d)** FACS-based approach (*n* = 3). **(e)** TO-PRO-3 and A5 staining of THP-1 apoptotic cells and sorted ApoBDs compared to unstained WAS, data generated from **(d)**. **(f)** Representative DIC microscopy showing ApoBDs purified by low-speed centrifugation approach alone or in combination with FACS-based approach. **(g)** Diameter (μm) of sorted ApoBDs generated from THP-1 monocytes (representative of 1 independent experiment, *n* = 3). **(h)** PI uptake by ApoBDs from THP-1 WAS, sorted ApoBDs and sorted ApoBDs exposed to hyperthermic treatment (*n* = 3). **(i,j)** Purity of ApoBDs isolated from apoptotic Jurkat T cells **(i)** and HUVEC **(j)** WAS by FACS-based approach (*n* = 3). Error bars represent s.e.m. (*n* = independent experiment). Statistical significant differences determined by two-tailed *t*-test. ns, not significant. **P < 0.01.

**Figure 2:**
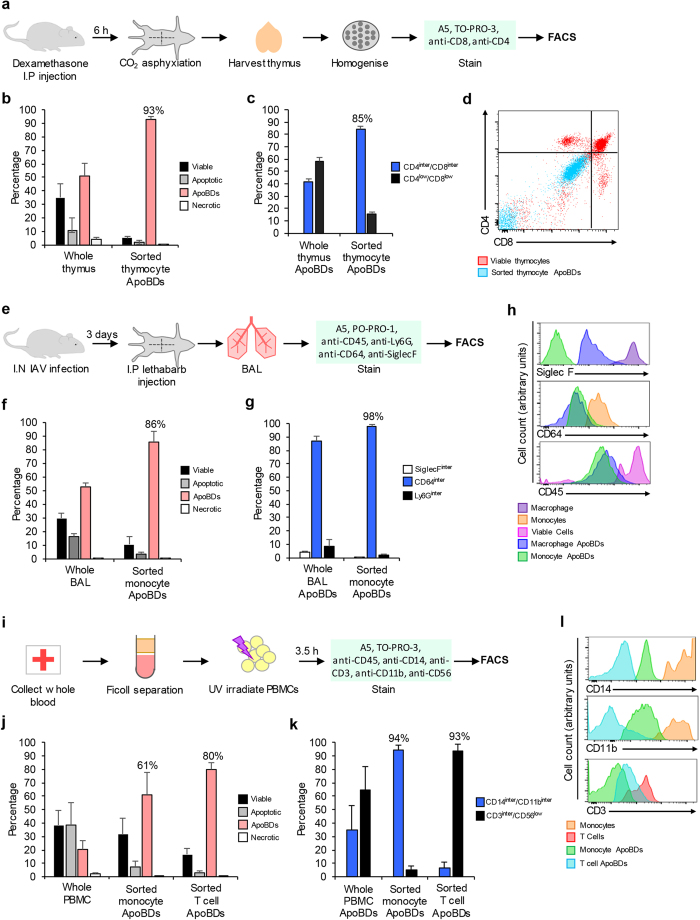
Cell type-specific ApoBDs can be identified and isolated from complex tissue, bodily fluid and blood-derived samples. **(a)** Schematic diagram of the dexamethasone mouse model used to generate and purify thymocyte-derived ApoBDs. **(b)** Purity of sorted thymocyte ApoBDs compared to whole thymus sample from dexamethasone-treated mice (*n* = 3). **(c)** Percentage of ApoBDs expressing intermediate CD4/CD8 and low CD4/CD8 from whole thymus or sorted ApoBD samples (*n* = 3). **(d)** CD4 and CD8 staining of viable thymocytes and sorted thymocyte ApoBDs. **(e)** Schematic diagram of the IAV mouse model used to generate and isolate BAL-derived monocyte ApoBDs. **(f)** Purity of sorted monocyte ApoBDs compared to whole BAL sample from IAV-infected mice (*n* = 3). **(g)** Percentage of ApoBDs expressing intermediate levels of SiglecF, CD64 or Ly6G from whole BAL or sorted ApoBD samples (*n* = 3). **(h)** Levels of SiglecF, CD64 and CD45 staining on macrophages, macrophage-derived ApoBDs, monocytes, monocyte-derived ApoBDs and total viable cells. **(i)** Schematic diagram of the PBMC model used to generate and purify human monocyte and T cell-derived ApoBDs. **(j)** Purity of sorted monocyte and T cell ApoBDs isolated from apoptotic PBMC samples (*n* = 3). **(k)** Percentage of ApoBDs expressing intermediate CD14/CD11b and intermediate CD3/low CD56 from whole PBMC, sorted monocyte and sorted T cell ApoBD samples (*n* = 3). **(l)** Levels of CD14, CD11b and CD3 expression on monocytes, monocyte ApoBDs, T cells and T cell ApoBDs. Error bars represent s.e.m. (*n* = independent experiment).
