# Supplementary material for: Isolation of cell type-specific apoptotic bodies by fluorescence-activated cell sorting
Source: Sci Rep. 2017 Jan 6;7:39846. doi: 10.1038/srep39846 (PMC5216387; doi:10.1038/srep39846)
Supplement: Supplementary Information [file srep39846-s1.pdf]

Isolation of cell type-specific apoptotic bodies by fluorescence-activated cell sorting

Georgia K Atkin-Smith, Stephanie Paone, Damien J Zanker, Mubing Duan, Than K Phan, Weisan Chen, Mark D Hulett & Ivan K H Poon.

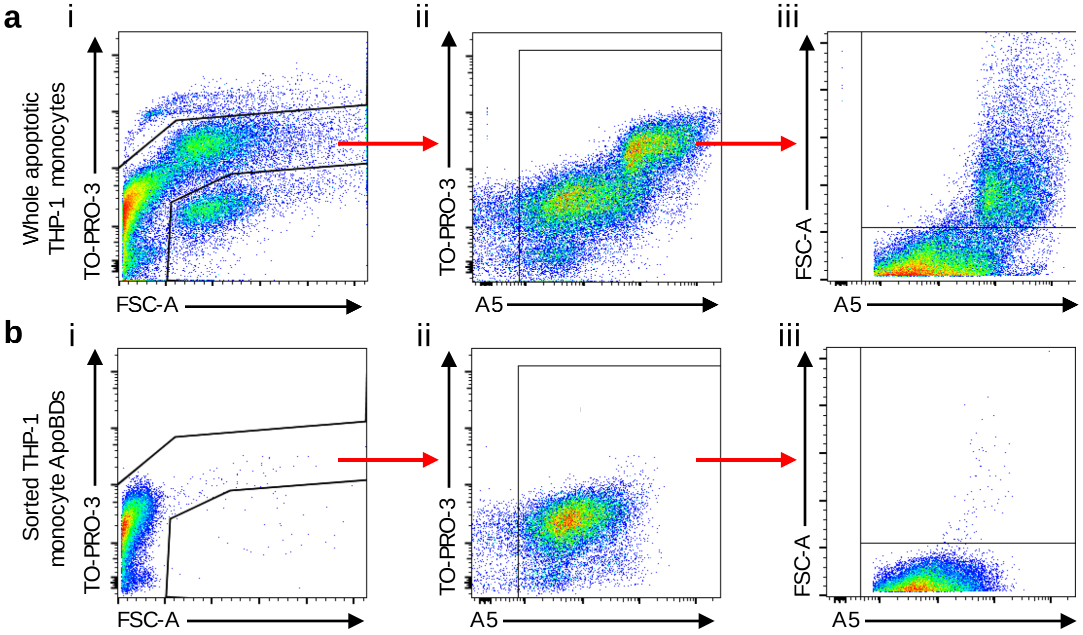

Supplementary figure 1. Electronic gating strategy used to isolate cell culture-derived ApoBDs. **(a)** THP-1 monocyte WAS analysis. (i) TO-PRO-3 high (i.e. necrotic cells) and TO-PRO-3 low and FSC intermediate/high (i.e. viable cells) events were separated from other events. (ii) A5 low (i.e. debris) events were separated from A5 intermediate/high events (i.e. apoptotic cells and ApoBDs). (iii) FSC intermediate/high (i.e. apoptotic cells) and FSC low (i.e. ApoBDs) events were separated. **(b, i-iii)** Re-analysis of sorted ApoBDs derived from THP-1 monocytes after isolation by FACS-based approach.

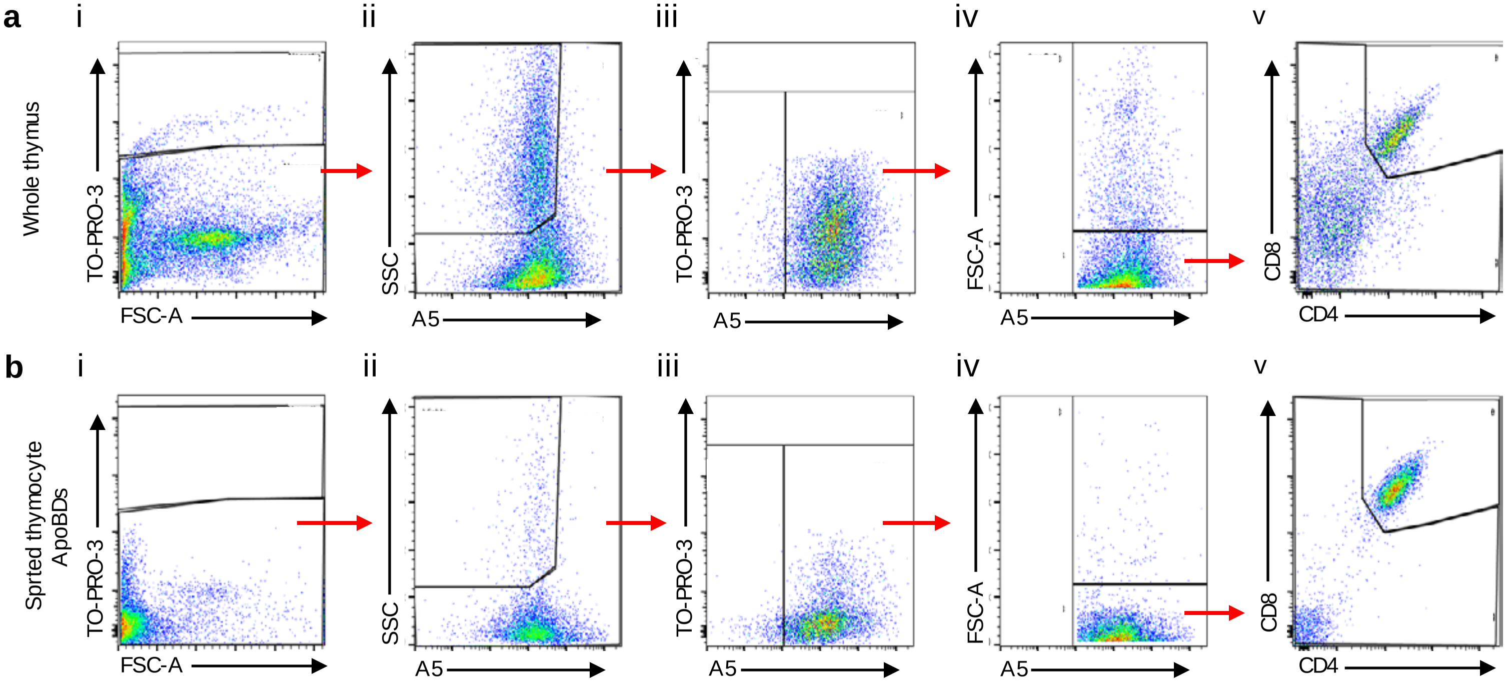

Supplementary figure 2. Electronic gating strategy used to isolate thymocyte-derived ApoBDs from whole thymus. **(a)** Whole thymus analysis. (i) TO-PRO-3 high (i.e. necrotic cells) events were separated from other events. (ii) SSC intermediate/high and A5 low (i.e. viable cells) were separated from other events. (iii) A5 low (i.e. debris) events were separated from A5 intermediate/high events (i.e. apoptotic cells and ApoBDs). (iv) FSC intermediate/high (i.e. apoptotic cells) and FSC low (i.e. ApoBDs) events were separated. (v) CD4 intermediate and CD8 intermediate (i.e. thymocyte-derived ApoBDs) events were separated from other events. **(b, i-v)** Re-analysis of sorted thymocyte-derived ApoBDs after isolation by FACS-based approach.

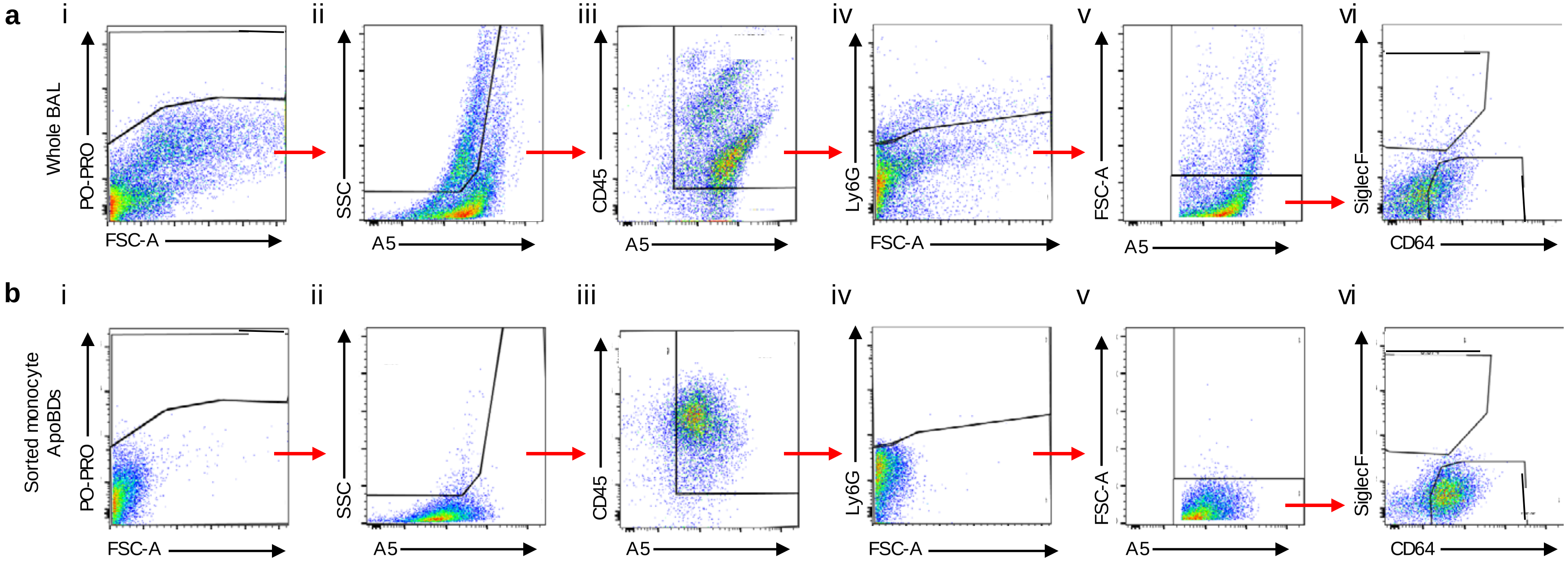

Supplementary figure 3. Electronic gating strategy used to isolate monocyte ApoBDs from whole BAL. **(a)** Whole BAL analysis. (i) PO-PRO-1 high (i.e. necrotic cells) events were separated from other events. (ii) SSC intermediate/high and A5 low (i.e. viable cells) were separated from other events. (iii) A5 low and CD45 low events were separated from other events. (iv) Ly6G high (i.e. neutrophils) events were separated from other events. (v) FSC intermediate/high (i.e. apoptotic cells) and FSC low (i.e. ApoBDs) events were separated. (vi) ApoBDs were separated into SiglecF intermediate (i.e. macrophage-derived ApoBDs) and CD64 intermediate (i.e. monocyte-derived ApoBDs) events. **(b, i-vi)** Re-analysis of sorted monocyte-derived ApoBDs after isolation by FACS-based approach.

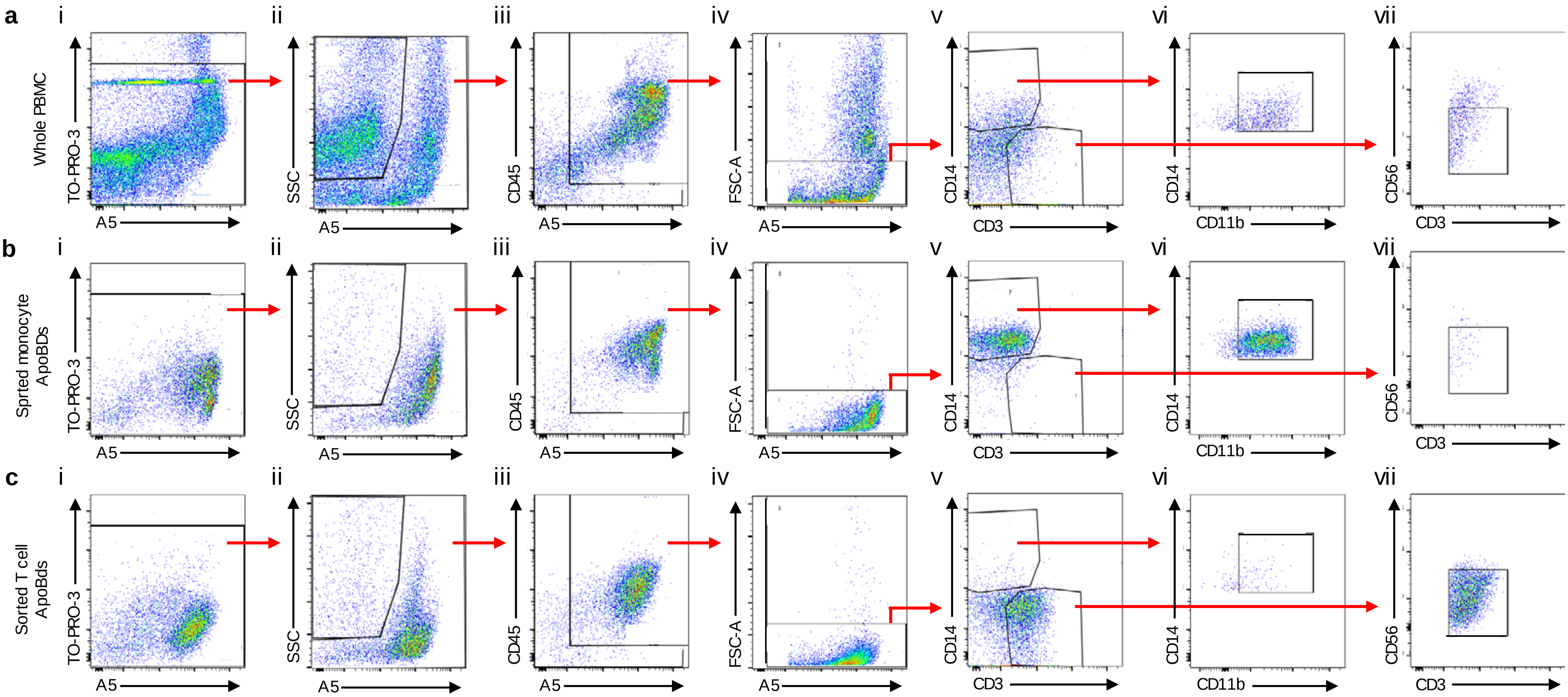

Supplementary figure 4. Electronic gating strategy used to isolate monocyte and T cell-derived ApoBDs from whole PBMCs. **(a)** Whole PBMC analysis. (i) TO-PRO-3 high (i.e. necrotic cells) events were separated from other events. (ii) SSC intermediate/high and A5 low (i.e. viable cells) were separated from other events. (iii) A5 low and CD45 low events were separated from other events. (iv) FSC intermediate/high (i.e. apoptotic cells) and FSC low (i.e. ApoBDs) events were separated. (v) ApoBDs were separated into CD14 intermediate and CD3 intermediate events. (vi) CD14 intermediate and CD11b intermediate (i.e. monocyte-derived ApoBDs) events separated from other events. (vii) CD3 intermediate and CD56 low (i.e. T cell-derived ApoBDs) events separated from other events. **(b,c, i-vii)** Re-analysis of sorted monocyte-derived ApoBDs **(b)** and T cell-derived ApoBDs **(c)**.
